# 

**Published:** 1895-01

**Authors:** 


CONTENTS—JANUARY, 1895.—VOL. X., NO. 7.

ORIGINAL COMMUNICATIONS—
Cerebral Syphilis. R. H. A. Boyd, M. D., San Antonio . . . . . . 325
Operative Procedures in Glandular Tumors of the Neck. Prof. J. E. Thompson, M. D., B. S., London Univ.; F. R. C. S., England . . 331
The Mirror Test for Nasal Obstruction. Prof. H. W. Loeb, A. M., M. D., St. Louis . . . . . . . . . . . . . . . . . . . . . . . . 338
Some Comments upon Erichsen's "Concussion of the Spine." S. C. Red, M. D., Houston.. . . . . . . . . . . . . . . . . . . . . 343

CORRESPONDENCE—
Better Organization in Medicine. L. D. Hill, M. D., Webberville . . 348
Medical Legislation in Texas. L. S. Downs, M. D.. . . . . . . . . . 350

EDITORIAL—
Texas' "One Man" Health Department. . . . . . . . . . . . . . . . 351
Texas Quarantine Expenses . . . . . . . . . . . . . . . . . . . . 353
The Appointments . . . . . . . . . . . . . . . . . . . . . . . . 354
Dignity and Impudence . . . . . . . . . . . . . . . . . . . . . . 357
Great Men Differ . . . . . . . . . . . . . . . . . . . . . . . . 359
Reductis ad Absurdum.. . . . . . . . . . . . . . . . . . . . . . 369

MEDICAL NEWS AND MISCELLANY—
Deadly Oysters, Medical Legislation, Ring out the Old Year, Vital Statistics, Contagious Diseases, Antitoxine in America, and numerous other Notes . . . . . . . . . . . . . . . . . . . . 361 to 368
Death of Prof. Sim . . . . . . . . . . . . . . . . . . . . . . . 369

CURRENT MEDICAL LITERATURE—
Department of Therapeutics. David Cerna, M. D., Galveston—Quinine in Cholera, Heart Disease, Antipyrin, Strontium Salicylate, Serum Treatment of Diphtheria . . . . . . . . . . . . . . . 369 to 372
BOOK NOTICES— . . . . . . . . . . . . . . . . . . . . . . . . 373 to 378
PUBLISHERS' NOTES . . . . . . . . . . . . . . . . . . . . . . . 378

